# Disposal practice and determinants of unused medicines among the general public in Gondar City, Northwest Ethiopia

**DOI:** 10.3389/fpubh.2025.1516809

**Published:** 2025-02-27

**Authors:** Lamrot Yohannes, Addisu Afrassa Tegegne, Gebremariam Genet, Liknaw Workie Limenh, Abdulwase Mohammed Seid, Tekletsadik Tekleslassie Alemayehu, Wondim Ayenew, Wudneh Simegn

**Affiliations:** ^1^Department of Environmental and Occupational Health and Safety, Institute of Public Health, College of Medicine and Health Sciences, University of Gondar, Gondar, Ethiopia; ^2^Department of Pharmaceutical Chemistry, School of Pharmacy, College of Medicine and Health Sciences, University of Gondar, Gondar, Ethiopia; ^3^Department of Pharmaceutics, School of Pharmacy, College of Medicine and Health Sciences, University of Gondar, Gondar, Ethiopia; ^4^Department of Clinical Pharmacy, School of Pharmacy, College of Medicine and Health Sciences, University of Gondar, Gondar, Ethiopia; ^5^Department of Social and Administrative Pharmacy, School of Pharmacy, College of Medicine and Health Sciences, University of Gondar, Gondar, Ethiopia

**Keywords:** disposal, determinants, unused medicines, Gondar, Northwest Ethiopia

## Abstract

**Introduction:**

The inappropriate disposal of unused medicines poses significant risks to public health and the environment. Gondar City, located in Northwest Ethiopia, is not exempt from this problem. However, little is known about the current disposal practices and the determinants influencing those practices in this specific context.

**Objectives:**

This study aimed to evaluate the disposal practices of unused medicines among the general public in Gondar City and to identify the factors that influence these practices.

**Methods:**

A cross-sectional study design was employed to collect data from the general public in Gondar City from July 30 to August 30, 2023. Data were collected through interviews using a structured questionnaire to gather information on disposal practices and determinants that could influence disposal practices.

**Results:**

A total of 845 respondents were involved in this study with a response rate of 93%. 56.9% of the respondents had poor disposal practices. Lower educational status [AOR = 2.76 (CI: 1.59, 4.79)]; *p* < 0.01, having a chronic disease [AOR = 1.79 (CI: 1.22, 2.64)]; *p* < 0.05, and poor knowledge toward disposal practices [AOR = 1.56 (CI: 1.12, 2.18)]; *p* < 0.05 were identified as significant determinants influencing poor disposal practices.

**Conclusion:**

There is an improper disposal practice of unused medicines in the study area. Lower educational status, having a chronic disease, and poor knowledge toward disposal practices were found to be significant factors. The finding underscores the need for targeted interventions to improve the safe disposal of unused medicines in Gondar City. Comprehensive educational campaigns should be launched to increase public awareness about the risks of improper disposal and promote safe disposal practices. The establishment of accessible and convenient disposal facilities or return programs should be prioritized.

## Background

According to the World Health Organization (WHO), unused medicines are defined as “medicinal products that have not been consumed by the patient, have not passed their expiry date, and have not been returned to a healthcare professional or a take-back program” (1). This definition includes medicines that remain unused by patients for reasons such as discontinuation of treatment, change in medication regimen, or medication surplus. It recommends returning unused medicines to a pharmacy or participating in a take-back program where available. If no take-back programs are available, the WHO suggests disposing of unused medicines in household waste, taking precautions to prevent accidental ingestion or misuse (1). The WHO advises against flushing unused medicines down the toilet or sinks to prevent potential contamination of water sources since medications contain harmful chemicals that may not be effectively removed through standard water treatment processes; hence why they recommend proper disposal methods to reduce environmental risks and protect public health (2, 3).

Globally, the improper disposal of unused medicine poses a danger to public health and environmental safety (4, 5). Different studies suggest that people use different methods for disposing of unused medicines, such as rinsing unwanted medicines down a sink, flushing them down the toilet, throwing them in the trash or garbage, or discarding them in household waste, which were some of the commonest disposal methods commonly practiced, contributing to environmental contamination, particularly in water bodies, soil, and wildlife (5–13). These disposal methods of unused medicines deviate from conventional disposal practices and indicate poor disposal practices (14, 15). This can lead to the presence of pharmaceutical compounds in aquatic ecosystems, causing harm to aquatic life and the environment and potentially entering the human food chain (15, 16). For example, studies have shown that trace amounts of pharmaceutical compounds found in water sources can disrupt the hormonal systems of aquatic species, including fish, leading to long-term ecological consequences (17). Additionally, improper disposal may result in the contamination of drinking water sources, posing significant risks to human health, such as the development of antibiotic resistance and exposure to harmful chemicals (18).

Improper disposal practices can vary across countries and communities, influenced by factors such as cultural norms, lack of awareness, and limited access to proper disposal facilities. Determining the specific factors or determinants that contribute to the accumulation of unused medicines can vary across contexts and populations. However, patient behavior and belief, prescription practice, medication adherence, availability of take-back programs, environmental concerns, and awareness were the common determinants of unused medicines in previous literatures (19–23). Moreover, studies suggest that individuals with lower educational status and limited access to health information are more likely to engage in poor disposal practices. Chronic diseases, which often result in the accumulation of unused medications, further exacerbate this issue, especially when patients lack clear guidance on how to dispose of their medications safely (20–23).

Given the increasing use of pharmaceuticals globally, it is essential to understand the determinants of poor disposal practices and develop tailored interventions. In countries like Ethiopia, where public awareness about pharmaceutical waste management is low, these practices are even more prevalent. There is limited research available on the disposal practices and determinants of unused medicines among the general public in Gondar City. This gap hinders the development of effective strategies to mitigate the risks associated with improper disposal and ensure safe medication management. Targeted educational programs and policy interventions, particularly in low- and middle-income countries, are necessary to mitigate the environmental impact and improve public health outcomes.

Therefore, this study aimed to assess the disposal practices of unused medicines and determinant factors among the general public in Gondar City. The findings of this study will not only contribute to the limited literature on disposal practices in Gondar City but will also provide valuable information for policymakers, healthcare providers, and relevant stakeholders to make targeted interventions that can be designed to improve public awareness, accessibility to proper disposal facilities, and regulatory frameworks.

## Methods

### Study design and period

The study utilized a cross-sectional study design and was conducted from June 30 to August 30, 2023. The municipal authorities reported a population of 454,445 people for the fiscal year 2021/2022. The town is organized into six sub-towns, namely Fasil, Zobil, Jantekel, Arada, Azezo, Tseda, and Maraki, and 36 Kebele administrations.

### Population

The source population for this study comprised all residents residing in Gondar City. The study population included all individuals residing in Gondar City, Northwest Ethiopia, who were eligible to participate in the study. Residents from selected kebeles who are heads or members of the household (age greater than 18 years) and willing to participate in the study were included in the study. Closed houses and newcomers in the selected kebele were excluded from the study.

### Sample size calculation and sampling

The sample size (n) of 845 was computed utilizing a single population proportion formula, by considering no previous study regarding the disposal of unused medicines in the area. This calculation incorporated a 95% confidence interval, a margin of error (d) set at 5%, and an additional 10% for the non-response rate. A design effect was factored in by multiplying the result by 2.

$$n=\frac{{\left(z_{\frac{a}{2}}\right)}^{2}\times p\times \left(1-p\right)}{d^{2}}=\frac{{\left(1.96\right)}^{2}\times 0.05\times \left(1-0.05\right)}{{\left(0.05\right)}^{2}}+10\%=423$$

**Figure 1:**
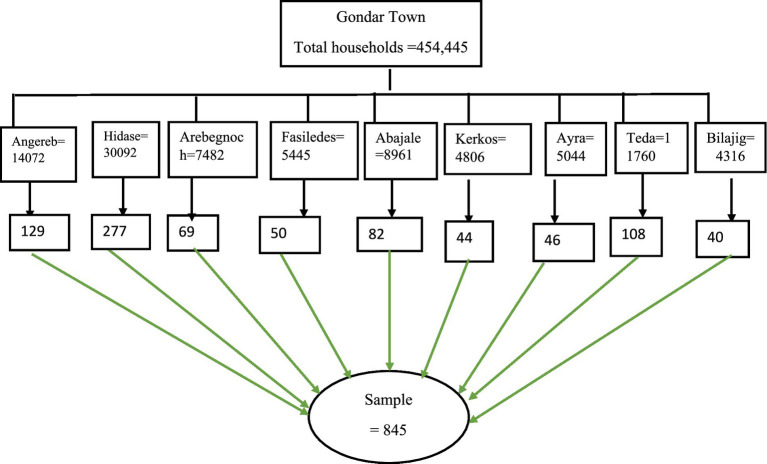
Schematic presentation of the sampling procedure for selecting study participants, Gondar Town, Northwest Ethiopia, 2023 (*n* = 845).

Gondar city encompasses 36 Kebeles distributed among six sub-city administrations. We specifically selected nine Kebeles (Angereb, Arebegnoch, Fasiledes, Abajale, Kerkos, Ayra, Teda, Bilajig, and Hidase) using a lottery method for inclusion in the study (Figure 1). The households were proportionately allocated to each kebele through the utilization of a stratified sampling technique. The list of households, along with their corresponding addresses, was acquired from the town administration and each Kebele administrative office. Within each stratum, samples were selected in proportion to their size, using the number of households as the sampling frame. A systematic random sampling technique was employed to select households from each Kebele. The sampling interval for each kebele was established by dividing the total number of households in each kebele by its proportionally allocated sample size. Subsequently, every Kth value of households was interviewed, with the initial household selected through a lottery method (Figure 1).

### Data collection tool and procedure

The questionnaire was adopted and modified from previously published similar studies (24–27) and validated through face-to-face validation by two pharmacy experts and one environmental health expert. The questionnaire had five parts. Part one included socio-demographic and socio-economic information about the study participants. Such as gender, age, residence, religion (orthodox, Muslim, Protestant, and others), marital status, educational status, occupation, and average monthly income were included in the socio-demographic part. Part two included 12 yes or no questions about knowledge of unused medicines disposal. Part three consists of ten five-point Likert scale questions on attitudes toward medication disposal. The fourth section of the questionnaire included eight yes or no questions about household drug storage practices among the public with and eight yes or no questions such as where they store their medications, whether the medication storage locations were locked, whether medicines kept in households have clear labels on their package material, whether they stored medications in a place at a height below the eye level of an average adult, do they give attention on the cold storage of refrigerated medicines, whether they check on the humidity of the medicines storage, do they give attention on keeping medicines away from the children and whether they give attention on eliminating medicines which had expired.

The last section of the questionnaire examined study participant’s disposal practice of unused medications such as whether a purchased medicine remain unused at their home (yes/no), do they have unused medicines because they stop taking the medicines when they feel better (yes/no), whether they dispose their medicines when experiencing unwanted side effects (yes/no), do they dispose their medicines when they smell bad, taste bad, or look bad (yes/no), whether they have unused medicines because they do not feel better as expected (yes/no), whether they keep medicines that they no longer require just in case they need them in the future (yes/no), do they throw waste unused medicine other than return to pharmacy (yes/no), whether they dispose of unused medicines by sharing/donating them tofriends (yes/no) and ever read medicines disposal instructions (yes/no).

The questionnaire was prepared in English and translated into the Amharic language for obtaining valid responses from the participants who cannot understand English. A bilingual expert performed a forward translation, and another bilingual expert independently performed the backward translation. The original and backward-translated questionnaires were discussed by the study team multiple times, and corresponding changes were made to the Amharic language question. Two pharmacy professionals were recruited to facilitate the data collection process, considering their previous experience.

### Variables of the study

The dependent variable was the disposal practices of unused medicines. Socio-demographic characteristics such as gender, age, residence, religion, marital status, educational status, occupation, and average monthly income and level of knowledge, attitudes toward medication disposal, and storage practices among the general public were the independent variables.

- Good disposal practice—Study participants were asked yes or no questions. 1 point was given for the correct answer, and 0 points were given for the incorrect answer. The sum of the value was 0 to 9. The mean value was taken as a cutoff point to categorize as good disposal practice and poor disposal practice. Those study participants who scored the mean and above the mean were considered as having good disposal practice (25).
- Poor disposal practice - Study participants who scored below the mean were considered as having poor disposal practice (25).
- Good knowledge—Individuals who had knowledge scores of at least eight out of the twelve questions were considered as having good knowledge (25, 28).
- Poor knowledge—Individuals who scored less than eight questions were considered as having poor knowledge (25, 28).
- Positive attitude toward medication disposal—those individuals who answered with “strongly agree” and “agree” to the questions were considered to have a positive attitude (25).
- Negative attitude toward medication disposal—those who answered with “strongly disagree” and “disagree” to the questions were considered to have a negative attitude (25).
- Good storage practice—those who answered ‘yes’ for the first question and ‘no for the left three questions were considered to have a good drug storage practice (25).
- Poor storage practice—those who answered differently from the answer result were considered to have a poor drug storage practice (25).

### Quality control

Data quality issues were assured by conducting a pretest among 50 study participants outside the study area among non-selected kebele. The questionnaires were checked for consistency, completeness, clarity, and accuracy. Minor modifications were made based on the findings of the pretest before the actual data collection. Training was given to data collectors about interview techniques, ethical issues, and data collection procedures. The data was checked from the beginning of the data collection to the end of the analysis. The first author has strictly followed all the processes to ensure the quality of the data.

### Statistical analysis

The collected data were entered and cleared using the EPI-INFO version 7.0.0 statistical package and exported to STATA version 17 for statistical analysis. Descriptive statistics such as frequencies, percentages, means, and standard deviations were employed. Bivariate and multivariable logistic regressions were employed to identify associated factors. Those independent variables with a *p*-value <0.2 were candidate variables for multiple logistic regression. Those variables with a *p*-value less than 0.05 were declared to have statistical significance.

## Results

### Socio-demographic characteristics of respondents

In terms of gender, 55.0% are males and 45.0% are females. The age distribution shows that 21.1% fall within the 19–25 age range and 29.0% are in the age range of 26–30. The majority of the respondents follow the Orthodox religion, accounting for 77.4%. Marital status reveals that 53.7% are married, and 1.5% are widowed. In terms of educational status, 12.5% are unable to read and write, and 46.1% have attained college-level or higher education. The majority of individuals live in rental accommodations (49.4%) or private houses (44.9%), with 4.7% cohabiting and 1.0% living in other types of residences (Table 1).

**Table 1:** Socio demographic characteristics among the general public in Gondar City (*n* = 786).

Variables	Categories	Frequency	Percent (%)
Gender	Male	432	55.0
	Female	354	45.0
Age in years	19–25	166	21.1
	26–30	228	29.0
	31–43	194	24.7
	44+	198	25.2
Religion	Orthodox	609	77.4
	Muslim	135	17.2
	Protestant	24	3.1
	Catholic	18	2.3
Marital status	Single	269	34.2
	Married	422	53.7
	Divorced	83	10.6
	Widowed	12	1.5
Educational status	Unable to read and write	98	12.5
	Primary (Grade 1–8)	106	13.5
	Secondary (Grade 9–12)	220	28.0
	College and above	362	46.1
Work	Employee	235	29.9
	Merchant	219	27.9
	Farmer	53	6.7
	House wife	113	14.4
	Student	42	5.3
	Retire	65	8.3
	No work	59	7.5
Living conditions	Private house	353	44.9
	Rental	388	49.4
	Cohabitant	37	4.7
	Others (given by Kebele and University)	8	1.0
Number of family	One	188	23.9
	Two	148	18.8
	Three	157	20.0
	Four	134	17.0
	Five and above	159	20.2
Monthly income (ETH-Birr)	0–4,000	245	31.2
	4,001–6,000	179	22.8
	6,001–8,500	188	23.9
	>8,500	174	22.1

### Disposal practice of unused medicines and related information

**Figure 2:**
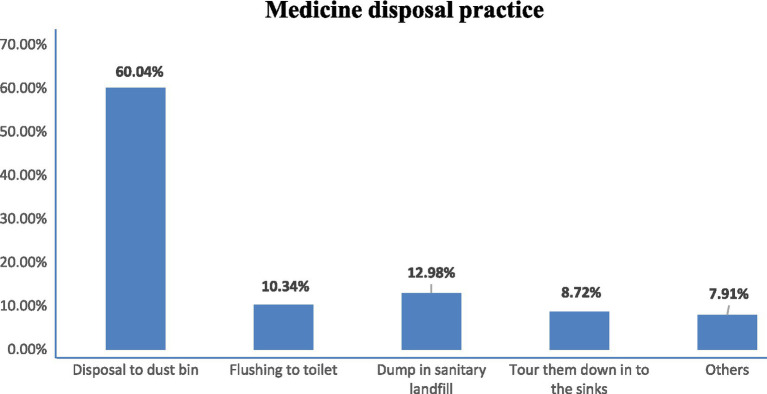
Disposal practice of study participants.

The most common disposal method for unused medicines was throwing them in the dustbin (60.04%), followed by dumping them into sanitary landfill (12.98%) (Figure 2).

From Table 2 below, the variable “Having chronic diseases” indicates that 24.3% of the population has chronic diseases. The “Smoking habit” variable shows that 9.0% of the population smokes, while 90.1% do not. In terms of receiving information about safe disposal of unused medicines, 23.8% have received such information. Among those who received information, the sources were pharmacy (8.8%), families and friends (5.7%), mass media (5.0%), physicians (1.3%), and the pharmaceutical industry (1.5%). In terms of health insurance coverage, 54.6% have health insurance, while 45.4% do not. When it comes to checking the expiry date of drugs during purchasing, 46.8% of the population does so, while 53.2% do not (Table 2).

**Table 2:** Drug disposal-related information among the general public in Gondar City (*n* = 786).

Variables	Categories	Frequency	Percent (%)
Having chronic diseases	Yes	191	24.3
	No	595	75.7
Smoking habit	Yes	71	9.0
	No	715	90.1
Ever received any information about safe disposing of unused medicines	Yes	187	23.8
	No	599	76.2
The source of information about safe disposing of unused medicines (More than one answer was possible)	Pharmacy	69	8,m.8
	Families and friends	45	5.7
	Mass media	39	5.0
	Physician	24	1.3
	Pharmaceutical industry	10	1.5
The source of medicines for the recently taken	Community pharmacy	485	61.7
	Public health facility	183	23.3
	Private health facility	102	13.0
	Friends/ Family	16	2.0
Having health insurance	Yes	429	54.6
	No	357	45.4
Checking expired date of drugs during purchasing	Yes	368	46.8
	No	418	53.2
Using any medicine in the previous 2 months	Yes	622	79.1
	No	164	20.9
Having unused medicine if using in the previous 2 months	Yes	432	69.4
	No	190	30.6
Reasons for unused medicine	Self-discontinuation after healing	188	43.5
	Anticipated future use	93	21.6
	Changed by health professionals	55	12.7
	Adverse effects	42	9.7
	Prescribed more than needed	35	8.1
Storage practice	Good	462	58.8
	Poor	324	41.2
Attitude toward disposal of unused medicine	Positive*	337	42.9
	Negative*	449	57.1
Knowledge toward disposal of unused medicine	Good	335	42.6
	Poor	451	57.4

*Positive attitude - an individual’s positive and constructive outlook, proactive approach, and willingness to engage with ideas, actions, or situations related to the disposal of unused medications, including adherence to safe and environmentally responsible disposal practices.

*Negative attitude - an individual’s disregard or indifferent perspective toward the proper disposal of unused medicines, including a lack of concern for the potential environmental and health consequences of improper medications disposal practices.

### Disposal practice of unused medicine

In the current study, about 56.9% of the respondents had poor disposal practices of unused medicines.

### Determinants of disposal practice of unused medicine

In the current study, gender, educational status, whether the participant ever received any information about safe disposal of unused medicines, whether they have a chronic disease, storage, practice, knowledge, and attitude were candidate variables for multiple logistic regression (*p* < 0.2). In the final model, educational status with cannot read and write (AOR = 2.76, 95% CI: 1.59, 4.79), having chronic disease (AOR = 1.79, 95% CI: 1.22, 2.64), and poor knowledge (AOR = 1.07, 95% CI: 0.77, 1.5) were significantly associated with poor disposal practice of unused medicines and considered as determinants of disposal practice of unused medicine (Table 3).

**Table 3:** Associated factors of medicine disposal practice among the general public in Gondar City (*n* = 786).

Variables	Categories	Disposal practice		COR (95% UI)	AOR (95% UI)
		Poor (%)	Good (%)		
Gender	Male	209 (53.0)	203 (47.0)	1	1
	Female	218 (61.6)	136 (38.4)	1.42 (1.07, 1.89)	1.22 (0.89, 1.66)
Educational status	Cannot to read and write	75 (76.5)	26 (23.5)	4.35 (2.61, 7.26)	2.76 (1.59, 4.79) **
	Primary school (1–8 grade)	87 (82.1)	19 (17.6)	2.26 (1.32, 3.87)	1.69 (0.95, 3.01)
	Secondary school (9–12 grade)	130 (59.1)	90 (40.9)	0.71 (0.36, 1.41)	0.53 (0.26, 1.08)
	College and above	155 (42.8)	207 (57.2)	1	1
Ever received any information about safe disposing of unused medicines	No	370 (61.8)	229 (38.2)	2.31 (1.68, 3.22)	1.05 (0.70, 1.56)
	Yes	77 (41.2)	110 (58.8)	1	1
Do you have chronic disease?	Yes	137 (71.7)	54 (28.3)	2.33 (1.64, 3.32)	1.79 (1.22, 2.64) *
	No	310 (52.1)	285 (47.9)	1	1
Storage practice	Good	246 (53.2)	216 (46.8)	1	1
	Poor	201 (62.0)	123 (38.0)	1.44 (1.07, 1.92)	1.31 (0.94, 1.82)
Knowledge	Good	153 (45.5)	183 (54.5)	1	1
	Poor	294 (65.3)	156 (34.7)	2.25 (1.68, 3.01)	1.56 (1.12, 2.18) *
Attitude	Positive	170 (50.4)	167 (49.6)	1	
	Negative	277 (61.7)	172 (38.3)	1.58 (1.19, 2.11)	1.07 (0.77, 1.49)

Hosmer and Lemeshow goodness of fit *p* = 0.751, *p < 0.05 and **p < 0.01.

## Discussion

The present study assessed disposal practice and determinants of unused medicines among the general public in Gondar city. The findings of this study revealed that 56.9% of the study participants had poor disposal practices, from a total of eight-hundred forty-five study participants with 93% response rate. These results suggested that a significant proportion of the population in Gondar City do not properly dispose of their unused medicines.

In this study, the proportion of respondents who have a good disposal practice (43.1%) is lower as compared to a study conducted in Northern Nigeria, which is 60.2% (27), in Tanzania (96.0%) (29), in Kenya (28), in Iraq (70%), in Jordan (58.1%) (31) and in Pokhara (Western Nepal) showing 50% of the study participants practicing proper drug disposal (24). This study finding also aligns with results from similar studies across various regions. For example, a study in Turkey and Northern Nigeria also revealed poor practices, where 81 and 60.2% of the study participants disposed by throwing them in the garbage, indicating widespread improper disposal behaviors for unused and expired medications (30, 31). Moreover, another systematic review found that urban households, particularly in Asia and Africa, demonstrated significant storage and wastage of medicines, with improper disposal being a common issue across regions (32). The variation in the prevalence of poor disposal practices for unused medicines across different studies and countries can be attributed to several factors. First, differences in healthcare systems and regulations might be the reason that countries with well-established healthcare systems and clear regulations on pharmaceutical disposal tend to have lower rates of improper disposal. For instance, high-income countries often have formalized take-back programs or designated drop-off locations for unused medicines, while lower-income countries may lack such infrastructure (32). In addition, public knowledge and awareness about proper medicine disposal significantly influence behavior. Differences in literacy levels and public health education programs play a crucial role. Moreover, regarding accessibility to proper disposal facilities and socioeconomic factors, higher-income countries tend to have more resources for waste management, public health infrastructure, and environmental regulations. In contrast, in low- and middle-income countries, poor waste management systems and fewer regulations may contribute to higher rates of improper disposal of medicines (32). The improper disposal of unused medications can have significant implications for public health, including contributing to drug pollution and the development of antibiotic-resistant bacteria. When medications are disposed of inappropriately, either by flushing them down the toilet or throwing them in the regular trash, they can end up in the water supply or soil, leading to contamination and potential harm to human health. One major concern is the impact of pharmaceutical pollution on the environment, as it can contribute to the development of antibiotic-resistant bacteria and the spread of drug resistance in the community. A study by Larsson et al. highlighted the link between pharmaceutical pollution and the development of antibiotic resistance (33). Improper disposal of antibiotics, in particular, can lead to the release of active drug compounds into the environment, providing a selection pressure for bacteria to develop resistance. This can have serious implications for the effectiveness of antibiotics in treating bacterial infections and can pose a significant threat to public health (33).

In Gondar City, like many urban areas, pharmaceutical contamination in the environment is a growing concern due to various factors, including the limited technologies for pharmaceutical removal in wastewater treatment plants. Research shows that pharmaceutical residues are present in water bodies due to the limited effectiveness of conventional wastewater treatment processes in eliminating these compounds. Studies, such as one conducted in Spain, have detected pharmaceutical residues in treated wastewater effluents, indicating the need for more efficient removal methods (34). Similarly, research by Kasprzyk-Hordern et al. (35) in the UK underlines the importance of advanced treatment technologies like ozonation and activated carbon filtration to prevent the release of pharmaceutical compounds into the environment from wastewater effluents (36–38).

The current study revealed that the most common method of disposing of unused medicines was tossing them in the dustbin, followed by dumping them in sanitary landfills and flushing them down the toilet. This disposal practice was consistent with similar studies conducted in Malaysia (39–41). In contrast, studies in the United States, Canada, the United Kingdom, and Australia (42–44) showed a preference for take-back programs organized by pharmacies, healthcare facilities, or local governments for medication disposal. Interestingly, findings from Ghana and Afghanistan diverged from this study results, as burying unused medicines in the ground and storing them at home until they expired were identified as the predominant disposal practices in these countries, respectively (22, 45). These differing practices may be attributed to variations in awareness levels, laws and regulations regarding medication disposal practice, infrastructure availability, attitudes toward medication disposal, prevalent health conditions, and the effectiveness of local pharmacists, healthcare providers, and health offices in promoting safe disposal practices.

In the current study, factors such as lower educational status, having a chronic disease, and poor knowledge toward disposal practices were identified as being significantly associated with the poor disposal practices of unused medicines.

Based on our study, study participants who cannot read and write (AOR = 2.76, 95% CI: 1.59, 4.79) were 2.76 times more likely to have a poor disposal practice compared with individuals who had completed primary school, secondary school, or college and above. This finding aligns with previous research conducted in various countries, which has shown that higher education levels are often associated with better health-related practices, including medication disposal (46, 47). This is consistent with research from Egypt, Pakistan, and India, which highlighted that individuals with lower levels of education are less likely to understand and practice proper disposal methods (32, 48). It is hardly surprising that improved health practices, such as disposing of medications properly, are frequently linked with higher education levels. Higher-educated people may be more aware of their surroundings and more perceptive about information, but lower-educated people are more prone to encountering issues and may not be aware of the negative consequences of improper medicine disposal. While higher education levels may be associated with better knowledge of proper medication disposal methods, it is essential to consider that education alone does not determine an individual’s behavior in this regard. Factors such as awareness, access to information, and personal beliefs also influence how people choose to dispose of their medications. Moreover, the lack of awareness about existing laws and medication disposal programs presents a significant challenge that requires attention and resolution. In Ethiopia, there are waste management directives for medicine disposal that incorporate a drug take-back program (49). Despite the existence of these directives, there has been limited initiative to enhance public awareness regarding the safe disposal of unused medicines and allocate budgets to facilitate the disposal of unused medicines.

Poor knowledge (AOR = 1.07, 95% CI: 0.77, 1.49) is another significant factor in this study. Respondents with poor knowledge about how to dispose of unused drugs were 1.07 times more likely to dispose of pharmaceuticals improperly. This is supported by a study that found those with poor knowledge were 4.8 times more likely to improperly dispose of unused medications (50). Furthermore, in research conducted in Arba Minch (51), 82 % of the respondents said they had no prior information about how to properly dispose of medications, and the majority of respondents (*n* = 208; 59.7%) said that medications that were unused or expired were considered waste. In addition, a study conducted in Qatar and Southwest Ethiopia showed the majority of the households lack knowledge on safe disposal methods, and most disposal methods used by households are not recommended (52). Education plays a key role in developing the importance of medicines and treatment regimens, rational utilization of healthcare services, proper disposal methods and an understanding of the needs and demands of pharmaceutical care. Furthermore, knowledge also maximizes the opportunity to exercise better control over the appropriate disposal of drugs, treatment plans, medical problems, treatment regimens, and disease conditions.

Having chronic diseases was found to be significantly associated with poor disposal practices of unused medicines. Studies show that individuals with ongoing health conditions tend to accumulate more medicines, leading to increased waste and improper disposal (30, 32). This finding is corroborated by studies in countries like Brazil, where people with chronic conditions tend to accumulate unused medicines due to frequent prescriptions but often lack clear instructions on safe disposal. In the U.S.A., medicines used for chronic diseases such as hypertension, diabetes, nervous system, gastrointestinal disease, cholesterol and heart disease, pain, and mental health problems are commonly reported unused medicines, indicating that many patients with chronic diseases keep leftover medications, unaware of the risks associated with improper disposal (53). Contrary to what we found, respondents who took acute-sickness medications were more likely to discard any unused or leftover medication in a way that could be dangerous in Malaysia (41) and Arba Minch (51). This is because the duration of therapy for acute illnesses is often brief, and it is anticipated that patients will cease taking their prescription if their health improves. This leads to a rise in production as well as the unsafe disposal of unused medication. A possible explanation for the variance in chronic patients might be the current higher and rising prevalence of chronic health conditions and excessive pharmaceutical intake. A systematic review of observational studies across Ethiopia found that acute poisoning cases are more prevalent among individuals under 30 years of age, with a higher incidence in females. The substances frequently involved include organophosphates, household cleaning agents, and pharmaceuticals. The case fatality rate varies, with some studies reporting rates up to 14.8% (54). Farmers (18.8%), unemployed individuals (18.2%), students (54.3%) and daily laborers (13%) were identified as the predominant victims for the drug poisoning. Other groups were house wives, daily laborers, waitress, government employee, merchant, and prisoner (55, 56). Education level of the victims was reported were Illiterate (59.2%), primary school (21.4%), secondary school (16.5%), and higher institute (2.9%) level personnel (57). In relation with this, the prevalence of self-medication practice has persisted in both developing and developed countries. In previous studies in Gondar, the prevalence of self-medication practice was 50.2% in 2019 (58) and 79.2% in 2021 (59) and, more than 60% (60.9%) of the households had a history of self-medication practice within their family members in the town (58) which will influence the consumption and of disposal practice of medications. In addition to this self-medication habit, the COVID-19 pandemic has significantly influenced medication consumption and disposal practices worldwide, including in Ethiopia. During the pandemic, the public in Ethiopia exhibited a rise in self-medication habits as individuals sought to prevent or manage COVID-19 symptoms. People self-medicating, writing their own prescriptions, and improperly managing their medications have become more common, and lockdowns and restricted access to doctors have been related to this trend (60, 61). A study in Ethiopia found that medications such as antibiotics, vitamins (e.g., vitamins C and D), and traditional remedies were frequently used without proper medical consultation (62, 63). This increase in medication consumption could lead to a higher likelihood of unused medications in households and improper disposal.

### Limitations

Even if we offered a high level of accuracy, the study design and its implementation might have certain limitations. These include potential social desirability and self-reporting bias. Due to the cross-sectional nature of this study, it was impossible to determine a temporal link between the exposure and outcome variables. It is important to consider these limitations when interpreting the findings of the study.

## Conclusion

The current study’s findings revealed poor disposal practices of unused medicines in a significant proportion of the study participants. Low educational status, the presence of chronic diseases, and poor knowledge toward disposal practices were identified as significant determinants of poor disposal practices of unused medicines. Therefore, encouraging interventions include the implementation of educational programs that focus on safe disposal practices of unused medicines, considering individuals with chronic diseases and other target individuals with lower educational levels, emphasizing the importance of proper disposal and the potential risks, and providing reminders, and clear instructions on how to dispose of medicines safely, taking into account any specific considerations related to chronic conditions. Conducting further research to explore the cultural and contextual factors influencing disposal practices of unused medicines will also be very important. The stakeholders should also focus on the aforementioned factors to design appropriate intervention strategies.

## Data Availability

The dataset is accessible from the corresponding author upon reasonable request.

## References

[1] World Health Organization. Guidelines for safe disposal of unwanted pharmaceuticals in and after emergencies. Geneva: World Health Organization (1999).

[2] Tong AY Peake BM Braund R. Disposal practices for unused medications around the world. Environ Int. (2011) 37:292–8. doi: 10.1016/j.envint.2010.10.002, PMID: 20970194

[3] Wieczorkiewicz SM Kassamali Z Danziger LH. Behind closed doors: medication storage and disposal in the home. Ann Pharmacother. (2013) 47:482–9. doi: 10.1345/aph.1R706, PMID: 23535813

[4] NA D-ME Haight ME. Expert stakeholders’ views on the management of human pharmaceuticals in the environment. Environ Manage. (2006) 38:853–66. doi: 10.1007/s00267-005-0306-z, PMID: 16955232

[5] Harhay MO Halpern SD Harhay JS Olliaro PL. Health care waste management: a neglected and growing public health problem worldwide. Trop Med Int Health. (2009) 14:1414–7. doi: 10.1111/j.1365-3156.2009.02386.x, PMID: 19735368

[6] Gupta D Gupta A Ansari NA Ahmed QS. Patient’s opinion and practice toward unused medication disposal: a qualitative study. J Pharm Sci Innov. (2013) 2:47–50. doi: 10.7897/2277-4572.02574

[7] Abahussain EA Ball DE Matowe WC. Practice and opinion towards disposal of unused medication in Kuwait. Med Princ Pract. (2006) 15:352–7. doi: 10.1159/000094268, PMID: 16888392

[8] AlAzmi A AlHamdan H Abualezz R Bahadig F Abonofal N Osman M. Patients’ knowledge and attitude toward the disposal of medications. J Pharm. (2017) 2017:1–9. doi: 10.1155/2017/8516741, PMID: 29130019 PMC5654249

[9] Kuspis DA Krenzelok EP. What happens to expired medications? A survey of community medication disposal. Vet Hum Toxicol. (1996) 38:48–9. 8825752

[10] Trueman P Taylor DG Lowson K. Evaluation of the scale, causes and costs of waste medicines. Report of DH funded national project. New York and London: York Health Economics Consortium and the School of Pharmacy, University of London (2010).

[11] Arkaravichien W Ruchipiyarak T Thawinwan W Benjawilaikul S. A Threat to the Environment from practice of drug disposal in Thailand. EnvironmentAsia. (2014) 7:13–8. doi: 10.14456/ea.2014.3

[12] Osei-Djarbeng SN Larbi GO Abdul-Rahman R Osei-Asante S Owusu-Antwi R. Household acquisition of medicines and disposal of expired and unused medicines at two suburbs (Bohyen and Kaase) in Kumasi-Ghana. Innov Pharm. (2015) 4:85.

[13] Banwat SB Auta A Dayom DW Buba Z. Assessment of the storage and disposal of medicines in some homes in Jos north local government area of plateau state, Nigeria. Trop J Pharm Res. (2016) 15:989–93. doi: 10.4314/tjpr.v15i5.13

[14] Yang T du Y Sun M Meng J Li Y. Risk Management for Whole-Process Safe Disposal of medical waste: progress and challenges. Risk Manag Healthc Policy. (2024) 17:1503–22. doi: 10.2147/RMHP.S464268, PMID: 38859877 PMC11164087

[15] Jena M Mishra A Maiti R. Environmental pharmacology: source, impact and solution. Rev Environ Health. (2019) 34:69–79. doi: 10.1515/reveh-2018-0049, PMID: 30854834

[16] Alfian SD Khoiry QA Pratama MAA Wahyudin W Puspitasari IM Pradipta IS. Awareness and beliefs of community pharmacists on disposal of unused and expired household medications in Indonesia: a cross-sectional study. J Pharm Health Serv Res. (2023) 14:401–6. doi: 10.1093/jphsr/rmad043

[17] Boxall AB Rudd MA Brooks BW Caldwell DJ Choi K Hickmann S. Pharmaceuticals and personal care products in the environment: what are the big questions? Environ Health Perspect. (2012) 120:1221–9. doi: 10.1289/ehp.1104477 22647657 PMC3440110

[18] Narvaez JF Jimenez CC. Pharmaceutical products in the environment: sources, effects and risks. Vitae. (2012) 19:92–108. doi: 10.17533/udea.vitae.10865

[19] Kumirska JJM. Special issue “Pharmaceutical residues in the environment”. Molecules. (2020) 25:2941. doi: 10.3390/molecules25122941, PMID: 32604747 PMC7356860

[20] Kennedy J Tuleu I Mackay K. Unfilled prescriptions of Medicare beneficiaries: prevalence, reasons, and types of medicines prescribed. J Manag Care Pharm. (2008) 14:553–60. doi: 10.18553/jmcp.2008.14.6.553, PMID: 18693779 PMC10438235

[21] Alosaimi K Alwafi H Alhindi Y Falemban A Alshanberi A Ayoub N. Medication adherence among patients with chronic diseases in Saudi Arabia. Int J Environ Res Public Health. (2022) 19:10053. doi: 10.3390/ijerph191610053, PMID: 36011690 PMC9408114

[22] Bashaar M Thawani V Hassali MA Saleem F. Disposal practices of unused and expired pharmaceuticals among general public in Kabul. BMC Public Health. (2017) 17:1–8. doi: 10.1186/s12889-016-3975-z, PMID: 28061902 PMC5219664

[23] Gubae K Arega Moges T Agegnew Wondm S Bayafers Tamene F Kiflu M Aschale E. Ecopharmacology: knowledge, attitude, and medication disposal practice among pharmacy students. Integr Pharm Res Pract. (2023):185–93. doi: 10.2147/IPRP.S428457 37901480 PMC10612519

[24] Paudel E Choi E Shrestha N. Pharmaceutical waste management in private pharmacies of Kaski District, Nepal. Int J Innov Sci Res Technol. (2019) 4:2456–165.

[25] Ayele Y Mamu M. Assessment of knowledge, attitude and practice towards disposal of unused and expired pharmaceuticals among community in Harar city, Eastern Ethiopia. J Pharm Policy Pract. (2018) 11:1–7. doi: 10.1186/s40545-018-0155-9 30459955 PMC6236888

[26] Makki M Hassali MA Awaisu A Hashmi F. The prevalence of unused medications in homes. Pharmacy. (2019) 7:61. doi: 10.3390/pharmacy7020061, PMID: 31200530 PMC6631141

[27] Jha N Kafle S Bhandary S Shankar PR. Assessment of knowledge, attitude, and practice of disposing and storing unused and expired medicines among the communities of Kathmandu, Nepal. PLoS One. (2022) 17:e0272635. doi: 10.1371/journal.pone.0272635, PMID: 35925995 PMC9352092

[28] Angi’enda SA Bukachi SA. Household knowledge and perceptions on disposal practices of unused medicines in Kenya. J Anthropol Archaeol. (2016) 4:1–20. doi: 10.15640/jaa.v4n2a1

[29] Marwa KJ Mcharo G Mwita S Katabalo D Ruganuza D Kapesa A. Disposal practices of expired and unused medications among households in Mwanza, Tanzania. PLoS One. (2021) 16:e0246418. doi: 10.1371/journal.pone.0246418, PMID: 33539402 PMC7861449

[30] Köksoy S. Unused, expired pharmaceuticals and their disposal practices among the general public in Burdur-Türkiye: a cross-sectional study. BMC Public Health. (2024) 24:1303. doi: 10.1186/s12889-024-18788-0, PMID: 38741105 PMC11092099

[31] Salim I Jatau AI Lawal BK Haruna A Yunusa I Wada AS. Assessment of unsafe disposal of unused and expired medicines practices among households in north-West Nigeria. Niger J Pharm. (2022) 56:26. doi: 10.51412/psnnjp.2022.26

[32] Jafarzadeh A Mahboub-Ahari A Najafi M Yousefi M Dalal K. Medicine storage, wastage, and associated determinants among urban households: a systematic review and meta-analysis of household surveys. BMC Public Health. (2021) 21:1127. doi: 10.1186/s12889-021-11100-4, PMID: 34118923 PMC8196539

[33] Larsson DJ de Pedro C Paxeus N. Effluent from drug manufactures contains extremely high levels of pharmaceuticals. J Hazard Mater. (2007) 148:751–5. doi: 10.1016/j.jhazmat.2007.07.008, PMID: 17706342

[34] Gracia-Lor E Sancho JV Serrano R Hernández F. Occurrence and removal of pharmaceuticals in wastewater treatment plants at the Spanish Mediterranean area of Valencia. Chemosphere. (2012) 87:453–62. doi: 10.1016/j.chemosphere.2011.12.025, PMID: 22221664

[35] Kasprzyk-Hordern B Dinsdale RM Guwy AJ. The occurrence of pharmaceuticals, personal care products, endocrine disruptors and illicit drugs in surface water in South Wales, UK. Water Res. (2008) 42:3498–518. doi: 10.1016/j.watres.2008.04.026, PMID: 18514758

[36] Hu X-L Bao YF Hu JJ Liu YY Yin DQ. Occurrence of 25 pharmaceuticals in Taihu Lake and their removal from two urban drinking water treatment plants and a constructed wetland. Environ Sci Pollut Res. (2017) 24:14889–902. doi: 10.1007/s11356-017-8830-y, PMID: 28478598 PMC6677712

[37] Daniel W-B Jerker F Girma T Elias D Zinabu G. Pharmaceutical pollution in an Ethiopian Rift Valley Lake Hawassa: occurrences and possible ecological risks. Environ Chall. (2024) 15:100901. doi: 10.1016/j.envc.2024.100901

[38] Fekadu S Alemayehu E Dewil R van der Bruggen B. Pharmaceuticals in freshwater aquatic environments: a comparison of the African and European challenge. Sci Total Environ. (2019) 654:324–37. doi: 10.1016/j.scitotenv.2018.11.072, PMID: 30448654

[39] Ong SC Ooi GS Shafie AA Hassali MA. Knowledge, attitude and disposing practice of unused and expired medicines among the general public in Malaysia. J Pharm Health Serv Res. (2020) 11:141–8. doi: 10.1111/jphs.12333

[40] Ariffin M Zakili TST. Household pharmaceutical waste disposal in Selangor, Malaysia—policy, public perception, and current practices. Environ Manag. (2019) 64:509–19. doi: 10.1007/s00267-019-01199-y, PMID: 31399770

[41] Wang LS Aziz Z Chik Z. Disposal practice and factors associated with unused medicines in Malaysia: a cross-sectional study. BMC Public Health. (2021) 21:1–10. doi: 10.1186/s12889-021-11676-x, PMID: 34530791 PMC8447783

[42] Kelly F McMillan S Spinks J Bettington E Wheeler AJ. ‘You don’t throw these things out:‘an exploration of medicines retention and disposal practices in Australian homes. BMC Public Health. (2018) 18:1–12. doi: 10.1186/s12889-018-5753-6, PMID: 30119656 PMC6098630

[43] Thane G., (2021). A call to action: an evidence review on pharmaceutical disposal in the context of antimicrobial resistance in Canada, NCCID. Available at: . (https://nccid.ca/wp-content/uploads/sites/2/2021/03/A-Call-to-Action-An-Evidence-Review-on-Pharmaceutical-Disposal-in-the-Context-of-Antimicrobial-Resistance-in-Canada.pdf).

[44] Glassmeyer ST Hinchey EK Boehme SE Daughton CG Ruhoy IS Conerly O. Disposal practices for unwanted residential medications in the United States. Environ Int. (2009) 35:566–72. doi: 10.1016/j.envint.2008.10.007, PMID: 19081631

[45] Abruquah AA Drewry JA Ampratwum F. What happens to unused, expired and unwanted medications? A survey of a community-based medication disposal practices. Int J Dev Sustain. (2014) 3:2175–85.

[46] Shoaib M Raziq A Iqbal Q Saleem F Haider S Ishaq R. Disposal practices of unused and expired pharmaceuticals among the general public in Quetta city, Pakistan. PLoS One. (2022) 17:e0268200. doi: 10.1371/journal.pone.0268200, PMID: 35587932 PMC9119513

[47] Hendaus MA Darwish S Saleh M Mostafa O Eltayeb A al-Amri M. Medication take-back programs in Qatar: Parental perceptions. J Family Med Prim Care. (2021) 10:2697–702. doi: 10.4103/jfmpc.jfmpc_1141_20, PMID: 34568157 PMC8415692

[48] Shakeel S Wajiha I Hina R Shagufta N. Pharmacy students’ awareness and practices concerning the disposal of household medicines in Karachi, Pakistan. Lat Am J Pharm. (2021) 40:467–74.

[49] Food, Medicine and Healthcare Administration and Control Authority of Ethiopia. Medicines waste management and disposal directive. Addis Ababa: Food, Medicine and Healthcare Administration and Control Authority of Ethiopia (2011).

[50] Yimenu DK Teni FS Ebrahim AJ. Prevalence and predictors of storage of unused medicines among households in northwestern Ethiopia. J Environ Public Health. (2020) 2020:8703208. doi: 10.1155/2020/8703208 32300369 PMC7136802

[51] Asmamaw G Agedew T Tesfaye B Sasamo S Gena S Argeta M. Prevalence of leftover medicines, disposal practices, and associated factors in Arba Minch town, Southern Ethiopia. SAGE Open Med. (2023) 11:20503121231158214. doi: 10.1177/20503121231158214, PMID: 36935887 PMC10021103

[52] Feyissa D Siraj J. Knowledge, attitude and disposal practices of unused and expired medications among the general public in Mizan-Aman, Southwest Ethiopia. Health Sci. (2022) 11:1–13.

[53] Law AV Sakharkar P Zargarzadeh A Tai BWB Hess K Hata M. Taking stock of medication wastage: unused medications in US households. Res Social Adm Pharm. (2015) 11:571–8. doi: 10.1016/j.sapharm.2014.10.003, PMID: 25487420

[54] Chelkeba L Mulatu A Feyissa D Bekele F Tesfaye BT. Patterns and epidemiology of acute poisoning in Ethiopia: systematic review of observational studies. Arch Public Health. (2018) 76:1–10. doi: 10.1186/s13690-018-0275-3, PMID: 29988616 PMC6027736

[55] Desalew M Aklilu A Amanuel A Addisu M Ethiopia T. Pattern of acute adult poisoning at Tikur Anbessa specialized teaching hospital, a retrospective study, Ethiopia. Hum Exp Toxicol. (2011) 30:523–7. doi: 10.1177/0960327110377520, PMID: 20630913

[56] Chala TS Gebramariam H Hussen M. Two-Year Epidemiologic Pattern of Acute Pharmaceutical and Chemical Poisoning Cases Admitted to Adama Hospital Medical College, Adama, Ethiopia. Asia Pac J Med Toxicol. (2015) 4:106–11. doi: 10.22038/apjmt.2015.5978

[57] Teklemariam E Tesema S Jemal A. Pattern of acute poisoning in Jimma University specialized hospital, south West Ethiopia. World J Emerg Med. (2016) 7:290–3. doi: 10.5847/wjem.j.1920-8642.2016.04.009, PMID: 27942347 PMC5143314

[58] Jember E Feleke A Debie A Asrade G. Self-medication practices and associated factors among households at Gondar town, Northwest Ethiopia: a cross-sectional study. BMC Res Notes. (2019) 12:1–7. doi: 10.1186/s13104-019-4195-2 30890186 PMC6425615

[59] Kifle ZD Mekuria AB Anteneh DA Enyew EF. Self-medication practice and associated factors among private health sciences students in Gondar town, north West Ethiopia. A cross-sectional study. Inquiry. (2021) 58:00469580211005188. doi: 10.1177/00469580211005188, PMID: 33759621 PMC7995453

[60] Ayati N Saiyarsarai P Nikfar S. Short and long term impacts of COVID-19 on the pharmaceutical sector. Daru. (2020) 28:799–805. doi: 10.1007/s40199-020-00358-5 32617864 PMC7332346

[61] Elek P Bíró A Fadgyas-Freyler PJHE. Income gradient of pharmaceutical panic buying at the outbreak of the COVID‐19 pandemic. Health Econ. (2021) 30:2312–20. doi: 10.1002/hec.4378 34218496 PMC8420393

[62] Fetensa G Tolossa T Etafa W Fekadu G. Prevalence and predictors of self-medication among university students in Ethiopia: a systematic review and meta-analysis. J Pharm Policy Pract. (2021) 14:107. doi: 10.1186/s40545-021-00391-y, PMID: 34915938 PMC8679998

[63] Kahssay SW Hammeso WW Getachew D Woldeselassie BD. Prevalence and determinants of household medication storage during the COVID-19 outbreak in Southwest Ethiopia. Drug Healthc Patient Saf. (2023) 15:1–11. doi: 10.2147/DHPS.S392564 36699285 PMC9869910

