# Supplementary material for: Disposal practice and determinants of unused medicines among the general public in Gondar City, Northwest Ethiopia
Source: Front Public Health. 2025 Feb 27;13:1516809. doi: 10.3389/fpubh.2025.1516809 (PMC11904836; doi:10.3389/fpubh.2025.1516809)
Supplement: Supplementary file 1 [file Table_1.docx]

# Tool

| **Part 1. Socio-demographic data** | | | |
| --- | --- | --- | --- |
| Serial No | Question | Response |  |
| 1.2 | 101. sex 1. male 2. Female | ……………………. |  |
| 1.3 | 102. age | 1. Male 2. Female |  |
| 1.4 | 103. Marital status | 1. Single 2. Married 3. Divorced 4. Widowed 5. Living separately |  |
| 1.5 | 104. Educational status | 1. Can’t read and write 2. Primary (1-8) 3. Secondary (9-12) 4. college or above |  |
| 1.6 | 105. Occupation | 1. Housewife 2. Merchant 3. employee 4. Others |  |
| 1.7 | 106. Living condition | 1. Private 2. Rental 3. Cohabitant 4. Other, please specify |  |
|  | 107. Chronic illness  ***(Multiple answers are possible)*** | 1. Hypertension 2. Diabetes 3. Asthma 4. Rheumatoid arthritis 5. 2. Gout 6. Others (specify)_ |  |
|  | 108. Smoking status? | 1. No smoker 2. Smoker |  |
| 1.8 | 109. Number of family | 1. Only one member 2. Two members 3. Three members 4. Four members 5. Five members or above |  |
| 1.9 | 110. Monthly income | …………………. |  |
|  | 111. Have you ever received any information about safe way of disposing of unwanted pharmaceutical? | 1. Yes 2. No |  |
|  | 112. If yes, source of information | 1. Mass-media 2. Physician 3. Pharmacy 4. The pharmaceutical industry 5. Others |  |
|  | 113. Where did you get the medications? | 1. Government hospital or clinic 2. Private hospital or clinic 3. Retail/community pharmacy |  |
|  | 114. Are you currently covered by some form of health insurance or health plan? | 1. Yes 2. No |  |
|  | 115. Checking of Expiry dates at the time of purchasing | 1. Yes 2. No |  |
| **Part 2. Knowledge about unused medicines disposal** | | | |
| 2.1 | 201. Do you know about medication waste? | 1. Yes 2. No |  |
| 2.5 | 202. Did you know that improper medication disposal could harm the environment and population health? | 1. Yes 2. No |  |
|  | 203. Inappropriate medicine disposal causes accidental swallow by children? | 1. Yes 2. No |  |
|  | 204. Donating or sharing unused medicines minimizes or controls the hazardous effect of unused and expired medicines? | 1. Yes 2. No |  |
|  | 205. keeping unused medication in the home can be accidental use or misuse by someone else in the Household | 1. Yes 2. No |  |
|  | 206. Do you know about “drug-take-back system”? | 1. Yes 2. No |  |
|  | 207. The acceptable drug disposal method is disposing them to municipality garbage at your home. | 1. Yes 2. No |  |
|  | 208. Do you know that misused/repeated change or not complete antibiotics may cause drug resistance? | 1. Yes 2. No |  |
|  | 209. It is acceptable to dispose solid  medicines (such as tablets, capsules and patches) in the garbage | 1. Yes 2. No |  |
|  | 210. It is acceptable to dispose medicines  by flushing down the toilet | 1. Yes 2. No |  |
|  | 211. it is acceptable to return  unused medicines to a local pharmacy  or healthcare facility | 1. Yes 2. No |  |
|  | 212. It is acceptable to dispose creams  and ointments in the garbage | 1. Yes 2. No |  |
| **Part 3. Disposal practice of unused medications** | | | |
| 3.1 | 301. Did any quantity of purchased medicine remain unused at your home? | 1. Yes 2. No |  |
|  | 302. I have unused medicines because I stop taking the medicines when I  feel better | 1. Yes 2. No |  |
|  | 303. I dispose my medicines when I experience unwanted side effects | 1. Yes 2. No |  |
|  |  | 1. Yes 2. No |  |
|  | 304. I dispose my medicines when they smell bad, taste bad, or look bad | 1. Yes 2. No |  |
|  | 305. I have unused medicines because I do not feel better as I have  Expected | 1. Yes 2. No |  |
|  | 306. I keep medicines that I no longer require just in case I need them  in the future | 1. Yes 2. No |  |
|  | 307. I throw waste unused medicine other than return to pharmacy | 1. Yes 2. No, I return to pharmacy 3. I don’t know |  |
|  | 308.I disposeof unused medicines by sharing/donating them tofriends | 1. Yes 2. No |  |
|  | 309. Have you ever read medicines disposal instructions? | 1. Yes 2. No |  |
| 3.2 | If yes, where you put?   1. Dust bin 2. Flash them down the toilet 3. Tour them down into the sink 4. Dump in sanitary land fill 5. Return to Pharmacy/supplier | | |
| **Part 4. Attitudes toward disposal of medications** | | | |

| **Perception questions** | **Strongly agree** | **Agree** | **Neutral** | **Disagree** | **Strongly disagree** |
| --- | --- | --- | --- | --- | --- |
| 401. Unused medicines present potential risks at home? |  |  |  |  |  |
| 402. There is lack of adequate information on safe disposal of unused medicine |  |  |  |  |  |
| 403. Children are more vulnerable to the risks of associated with unused and expired household medicines |  |  |  |  |  |
| 404. Doctors and healthcare professionals do provide advice on safe disposal of unused and expired household medicines |  |  |  |  |  |
| 405. Take-back programs of unused and expired medicines should be mandatory |  |  |  |  |  |
| 406. Outreach and awareness programs about how to dispose unused or expired medicines should be initiated |  |  |  |  |  |
| 407. Prescription medication should be disposed of differently than over-the-counter or non-prescription medication? |  |  |  |  |  |
| 408. It is my responsibility to protect my household members from  unintended harmful exposure to unused medicines |  |  |  |  |  |
| 409. People should not flush unwanted prescription or non-prescription medications down the toilet or the sink? |  |  |  |  |  |
| 210. Would you use location where unused medications could drop off for disposal to get rid of such medications? |  |  |  |  |  |

| **Part 5. Assessment of house hold drug storage practice** | | |
| --- | --- | --- |
|  | 600. Where do you store your medications? | 1. In refrigerator 2. In a bathroom 3. In the kitchen 4. In the bedroom 5. In a purse or briefcase 6. At any other place |
|  | 601. Is the locations where medications stored locked? | 1. Yes 2. No |
|  | 602. Are medicines kept in households have clear labels on their package material? | 1. Yes 2. No |
|  | 603. Do you stored medications in a place at a height below the eye level of an average adult? | 1. Yes 2. No |
|  | 604. Did you pay your attention on the storage temperature of medicines? | 1. Yes   2. No |
|  | 605. Did you pay your attention on the cold storage of refrigerated medicines? | 1. Yes 2. No |
|  | 606. Did you check on the storage humidity of medicines? | 1. Yes 2. No |
|  |  |  |
|  | 607. Did you pay your attention on keeping medicines away from the children? | 1. Yes 2. No |
|  | 608. Did you pay your attention on eliminating medicines which had expired? | 1. Yes 2. No |
